# Localized Oxygen Exchange Platform for Intravital Video Microscopy Investigations of Microvascular Oxygen Regulation

**DOI:** 10.3389/fphys.2021.654928

**Published:** 2021-06-08

**Authors:** Richard J. Sové, Stephanie Milkovich, Hristo N. Nikolov, David W. Holdsworth, Christopher G. Ellis, Graham M. Fraser

**Affiliations:** ^1^Department of Biomedical Engineering, Johns Hopkins University School of Medicine, Baltimore, MD, United States; ^2^Department of Medical Biophysics, University of Western Ontario, London, ON, Canada; ^3^Robarts Research Institute, University of Western Ontario, London, ON, Canada; ^4^Division of BioMedical Sciences, Faculty of Medicine, Memorial University of Newfoundland, St. John's, NL, Canada

**Keywords:** microcirculation, blood flow, oxygen transport, microfluidics, capillary, oxygen saturation, mathematical modeling

## Abstract

Intravital microscopy has proven to be a powerful tool for studying microvascular physiology. In this study, we propose a gas exchange system compatible with intravital microscopy that can be used to impose gas perturbations to small localized regions in skeletal muscles or other tissues that can be imaged using conventional inverted microscopes. We demonstrated the effectiveness of this system by locally manipulating oxygen concentrations in rat *extensor digitorum longus* muscle and measuring the resulting vascular responses. A computational model of oxygen transport was used to partially validate the localization of oxygen changes in the tissue, and oxygen saturation of red blood cells flowing through capillaries were measured as a surrogate for local tissue oxygenation. Overall, we have demonstrated that this approach can be used to study dynamic and spatial responses to local oxygen challenges to the microenvironment of skeletal muscle.

## 1. Introduction

Oxygen (O2) regulation is a critical physiological function where precise regulatory control is required to ensure the metabolic demands of the tissues of the body are met (Duling, 1972; Sparks, 1980; Kontos and Wei, 1985; Golub and Pittman, 2013). In order for such a level of control to be possible, there must be various mechanisms in place to sense O2 demand and correspondingly adjust O2 supply. Numerous studies have confirmed that the presence or absence of O2 in the microcirculation results in a vasoactive response such that high levels of O2 result in vasoconstriction (Duling, 1972; Hutchins et al., 1974; Welsh et al., 1998; Zhu et al., 1998; Frisbee and Lombard, 2002) and low levels of O2 result in vasodilation (Pittman and Duling, 1973; Fredricks et al., 1994; Frisbee et al., 2002). These findings allude to the existence of an O2 sensor, the location of which remains unclear (Jackson, 2016). Potential locations include the red blood cell (RBC), arteriolar smooth muscle, arteriolar endothelium and even extra-vascular cells; see Jackson (2016) for an in-depth review.

There has been substantial evidence pointing to the RBC as the sensor for O2 in the microcirculation; see review by Ellsworth et al. (2009). One potential mechanism that has been proposed is the O2-dependent release of ATP from RBCs. In this mechanism, ATP is released from RBCs in response to decreased oxyhemoglobin saturation leading to increased plasma ATP (Bergfeld and Forrester, 1992; Ellsworth et al., 1995). The intra-luminal ATP can then bind to P2*Y*2 receptors on the blood vessel endothelium triggering an upstream vasodilatory response (Sprague et al., 1996; McCullough et al., 1997; Collins et al., 1998; Dietrich et al., 2000). Several pathological conditions have been associated with an impaired ability to release ATP, such as sepsis (Bateman et al., 2015) and type II diabetes (Sprague et al., 2006; Hanson et al., 2009; Ellis et al., 2010), potentially affecting the ability to regulate oxygen.

Various studies have used microscopy in conjunction with methods to alter the tissue oxygenation to interrogate the regulatory system (Welsh et al., 1998; Frisbee and Lombard, 2002; Frisbee et al., 2002). For instance, suffusion solutions with varying levels of O2 have been used to control O2 in several tissue preparations to study the regulatory response (Frisbee and Lombard, 2002; Frisbee et al., 2002). In previous studies, we used intravital video microscopy that combines a gas exchange platform with computer controlled gas flow meters to manipulate the gas composition at the surface of rat *extensor digitorum longus* (EDL) muscle to study the response of the microcirculation to a range of O2 concentrations (Jagger et al., 2004; Ellis et al., 2006; Milkovich et al., 2007). In these studies, the entire surface of the muscle was affected by the change in O2. While these approaches were able to elicit vasodilatory responses, more localized changes in O2 could potentially reveal information leading to the location of the O2 sensor.

More recently, a localized micro-delivery system was developed that was capable of limiting the change in RBC oxygen saturation (SO2) to a circular area of approximately 175 μm in diameter (Ghonaim et al., 2011), however, changes in RBC supply rate (SR) were not reported (Ghonaim et al., 2011). This finding was supported by a mathematical model of the regulatory system that suggests the signal for vasodilation is additive and depends on the number of capillaries that are stimulated (Ghonaim et al., 2013). A later study used a larger exchange window (1 mm long by 0.1 mm wide) to manipulate the RBC SO2 of a much larger area; this larger exchange window elicited a flow response (Ghonaim, 2013). This work further supports the idea that the vasodilatory signal is additive.

The work in Ghonaim (2013) showed promising results which were consistent with the proposed ATP release mechanism, however, there were some limitations for studying O2 regulatory mechanisms. First, stimulating multiple microvascular units at the same time potentially affects multiple feeding arterioles. Additionally, the setup in Ghonaim et al. could only resolve capillaries that were less than 60 μm from the surface; one challenge associated with using gas exchange chambers with intravital microscopy is that the chamber must be placed in between the objective and the muscle, reducing the focal depth to which the vasculature can be resolved. This impedes the ability to focus on structures deeper in the tissue.

The objective of the present study was to develop and validate a modular gas exchange device capable of changing local tissue O2 tension in micro-scale volumes and thus manipulating oxygen saturations within the overlying capillaries. One potential benefit of such a device is to determine if stimulation of a small number of microvascular units is sufficient to elicit a flow response. By making the design modular, the device can be easily adjusted to suit different needs and available equipment. For example, the shape and size of the exchange surfaces can easily be changed. This design also aims to maximize the resolvable depth permitted by the microscope objective's working distance in order to visualize structures deeper in the tissue as well as allowing for recording of adjacent regions in the tissue. In addition, we used a graphical processing unit (GPU) accelerated computational model of oxygen transport to estimate O2 content in the tissue and the temporal affects of changing O2 in the chamber. Overall, we describe a novel modular gas exchange device for studying microvascular oxygen regulation *in vivo* in tissues that can be imaged using conventional inverted microscopes.

## 2. Methods

### 2.1. Gas Exchange Chamber Design and Fabrication

**Figure 1:**
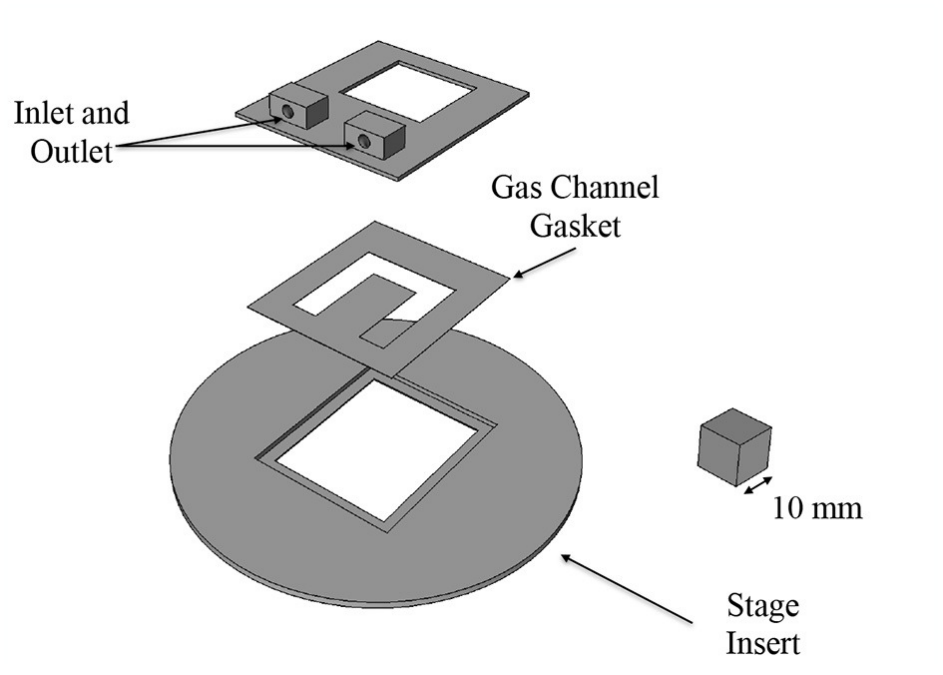
Three dimensional CAD model of gas chamber components. Inlet/outlet mount and stage insert were 3D printed. The gas channel gasket was made out of polymethyl-methacrylate (PMMA). The gas channel is sealed on the bottom with a glass coverslip and on the top with a glass coverslip patterned with laser-cut exchange windows.

The gas exchange chamber was comprised of a microscope stage insert, a gasket to form the side walls of the gas channel and a platform for the inlet and outlet of the channel (see Figure 1). The bottom of the channel was closed by a replaceable glass coverslip. The top of the channel was sealed by a custom, laser-cut 24 x 30 mm glass coverslip with five windows for gas exchange using a process described in Nikumb et al. (2005); the windows were mated with a thin, gas-permeable, membrane. The components were assembled together using vacuum grease to prevent gas leakage.

The stage insert and platform for the inlet and outlet were designed in FreeCAD and 3D printed. The gasket was fabricated by hand cutting 100 μm thick sheets of polymethyl-methacrylate (PMMA) to the desired shape. The gas-permeable membrane was fabricated in polydimethylsiloxane (PDMS) using a spin-coating technique similar to McDonald et al. (2000). PDMS (Sylgard 184, Dow Corning Corporation) in a 10:1 prepolymer to cross-linker ratio by weight was spin-coated at 1,700 rpm for 30 s; resulting membranes were approximately 25 μm in thickness.

Gas composition (O2, CO2, and N2) was controlled using computer controlled mass flow meters that imposed square wave oxygen oscillations within the exchange chamber consisting of 1 min of 5% O2 followed by 2 min of 12%, 2 min of 2%, and 1 min of 5% with static 5% CO2 and balance N2. Gas temperature was maintained at 37°C.

### 2.2. Animal Preparation

**Figure 2:**
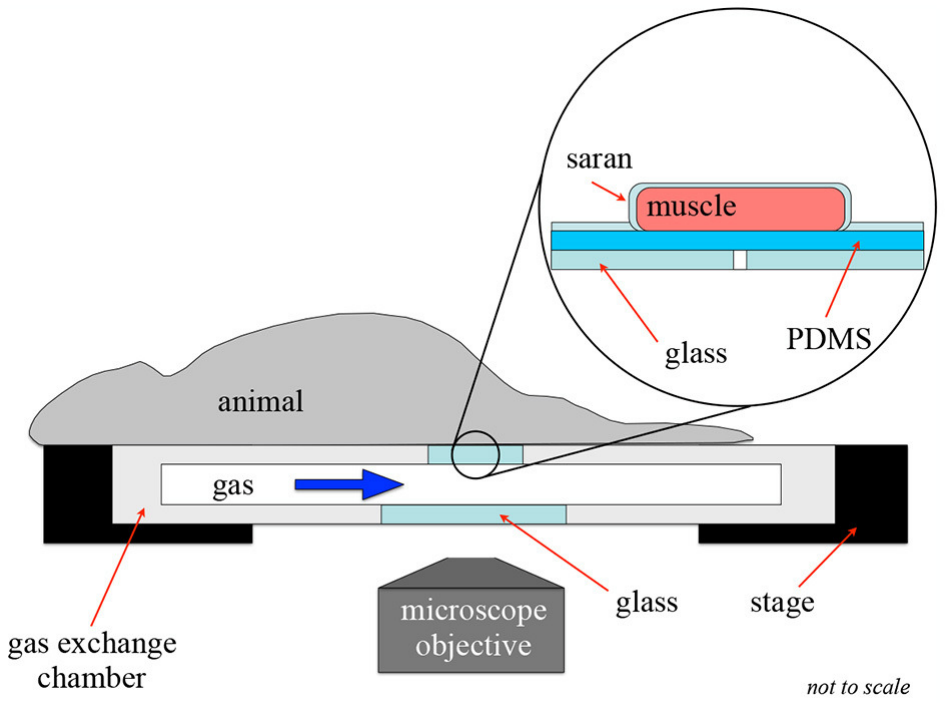
Intravital video microscopy experimental setup. This figure shows the experimental setup for an intravital experiment to allow for oxygen exchange to a localized area of tissue. In this setup, the muscle is extended over the gas exchange window and covered in gas-impermeable Saran wrap to isolate the muscle from the surrounding environment.

Four male Sprague-Dawley rats were allowed to acclimatize in animal facilities for 8–11 days prior to testing. Animals were group housed in a climate controlled room maintained between 21 and 24°C, with humidity of 25–40%, and a 12 h light/dark cycle. Animals were provided water and rat chow *ad libitum*. Animal weights ranged between 150 and 200 g on the day of testing. Rats were anesthetized with sodium pentobarbital (65 mg/kg) via intraperitonial injection. Prior to instrumentation, surgical plane of anesthesia was determined by the absence of palpebral, and toe pinch withrdrawal reflexes. Depth of anesthesia was repeatedly assessed over the duration of animal testing. Once surgical plane was achieved animals were instrumented with arterial and venous indwelling catheters and tracheotomized for ventilation as previously described (Ellis et al., 1992, 2010). Animals were mechanically ventilated with 30% oxygen and balance nitrogen while their inspired O2, heart rate, and blood pressure levels were continuously monitored to verify a normotensive state during data collection (mean arterial pressure > 70 mmHg) as described in Ellis et al. (2010). The *extensor digitorum longus* (EDL) muscle of the hind limb was prepared for microscopy as described in Fraser et al. (2012); this preparation was based on that by Tyml and Budreau (1991). The muscle was reflected on the 3D printed gas exchange chamber fitted into the microscope stage (Figure 2), and secured using suture attached to the distal tendon of the muscle. The muscle was then covered with oxygen-impermeable polyvinylidene film (Saran Wrap, Dow Corning) and a glass coverslip to isolate it from the room air. The tissue was trans-illuminated with a 75 W xenon lamp (Olympus U-LH75XEAPO) using an Olympus IX-81 inverted microscope equipped with 10X (Olympus U Plan S-APO; 0.4 NA) and 20X (Olympus U Plan LWD; 0.45 NA) objectives. The corresponding images were captured using the dual video camera system similar to that previously described in Arpino et al. (2017). During data collection, animal core temperatures were continuously monitored using a rectal thermocouple and maintained between 36 and 37 C using a heat lamp. Following data collection animals were euthanized with an injection of sodium pentobarbital (150 mg/kg) into the carotid artery cannula. The experiments used in this study were approved by the University of Western Ontario's Animal Care and Use Committee.

### 2.3. Data Analysis and Statistics

Intravital video microscopy images were acquired at 21 frames per second at two wavelengths, 438 and 450 nm using a beam splitter (Dual Cam 2, Photometrics) and two Rolera XR digital video cameras. Video sequences were processed to generate functional images (e.g., minimum intensity image) that define the location of flowing blood vessels due to the passage of red blood cells which strongly absorb light at the wavelengths selected (Japee et al., 2004). Images were analyzed offline using software developed in MATLAB (Mathworks) to quantify SO2 and hemodynamics via selection of individual capillaries in a semi automated fashion from functional images as described previously (Japee et al., 2004, 2005a,b; Fraser et al., 2012). SO2 and SR values for individual capillaries were binned every 5 s and presented as the mean of the bin to show representative responses in single vessels. Capillary RBC SO2, RBC SR, velocity, and hematocrit responses for all sampled capillaries were grouped based on proximity to the exchange window in the x-y plane (directly overlying the window, <100 μm from the window, 100–200 μm from the window, and >200 μm from the window) and statistical comparisons were made for each imposed gas exchange chamber oxygen concentration ([O2]). Capillary data sets were tested for deviation from normal distributions using Shapiro-Wilk tests, and Brown-Forsythe tests were used to evaluate differences in group variances. All normally distributed capillary data was paired across oxygen perturbations and repeated measures one-way analysis of variance (ANOVA) with Tukey's multiple comparison test was used to identify significant differences. Similarly, non-normally distributed capillary data was grouped by oxygen perturbations and Friedman tests with Dunn's multiple comparison post test was used to identify significant differences. A *p* value of < 0.05 was considered significant across all comparisons. All tests were performed using Prism 9 (Graphpad). Means and standard deviations are reported unless otherwise noted.

### 2.4. Mathematical Model of Tissue Oxygenation

A mathematical model of tissue oxygenation is used to determine the extent of oxygen diffusion in the tissue from the gas exchange chamber. Oxygen diffusion through tissue and the thin (~25 μm) PDMS barrier between tissue and the glass slide was modeled in 3D over time. Tissue oxygen partial pressure (PO2) was determined by numerically solving:

$$\begin{array}{l} k\frac{\partial P}{\partial t}=Dk\nabla^{2}P+Kk\left(1-\frac{P}{P_{0}}\right)-M_{0}\frac{P}{P+P_{\text{50}}},\in {\text{Ω}}_{\text{tissue}},\\ \;k^{\prime }\frac{\partial P}{\partial t}=D^{\prime }k^{\prime }\nabla^{2}P,\text{∈ }{\text{Ω}}_{\text{PDMS}}, \end{array}$$

where *D*/*D*′ and *k*/*k*′ are oxygen diffusivity and solubility in tissue/PDMS, respectively, *M*_0_ is the maximal tissue oxygen consumption, *P*_50_ is the PO2 at which consumption is half *M*_0_, *P*_0_ is the average capillary PO2 and *K* is the rate of oxygen transport from the capillaries into the tissue. Ω_tissue_ and Ω_PDMS_ represent the tissue and PDMS domains, respectively. The parameters used in our model are summarized in Table 1.

**Table 1:** Parameters used in mathematical model.

**Parameter**	**Value**	**References**
*D*	2.41 × 10^−5^ cm^2^/s	Bentley et al., 1993
*k*	3.89 × 10^−5^ mL O2/mL/mmHg	Mahler et al., 1985
*D*′	3.40 × 10^−5^ cm^2^/s	Merkel et al., 2000
*k*′	1.32 × 10^−5^ mL O2/mL/mmHg	Shiku et al., 2006
*M*_0_	1.57 × 10^−4^ mL O2/mL/s	Sullivan and Pittman, 1984
*K*	30 mmHg/s	Goldman, 2008
*P*_0_	48 mmHg	Goldman, 2008
*P*_50_	0.5 mmHg	Honig and Gayeski, 1982

This model assumes the tissue is homogeneously consuming oxygen and that there is a homogeneous supply of oxygen from the capillaries. Zero flux boundary conditions were specified for the tissue boundaries and along the glass surface. Fixed PO2 boundary conditions matching those employed in *in vivo* experiments were applied at the surface of the gas exchange window. Similar models were implemented in previous studies to predict tissue oxygenation (Goldman, 2008; Ghonaim et al., 2011). Our model also includes transport through the PDMS layer directly above the gas exchange window which was not incorporated in previous models.

The temporal derivative was discretized using an implicit-explicit method similar to Ascher et al. (1995) and the spatial derivatives were discretized using a second order central difference scheme. In this scheme, the linear source term was evaluated at the current time step, where as the other terms were evaluated at the previous time step. This scheme was chosen since it is fully explicit and has greater stability than the forward Euler scheme. The numerical solution was parallelized on a GPU and implemented in C++/CUDA. The numerical grid was spatially decomposed onto a 1024-core GPU.

We quantified the extent of the O2 perturbation in each dimension by calculating distance from the edge window in which the directional derivative of the PO2 is less than *e*^−4^ (≈0.02) mmHg/μm.

## 3. Results

**Figure 3:**
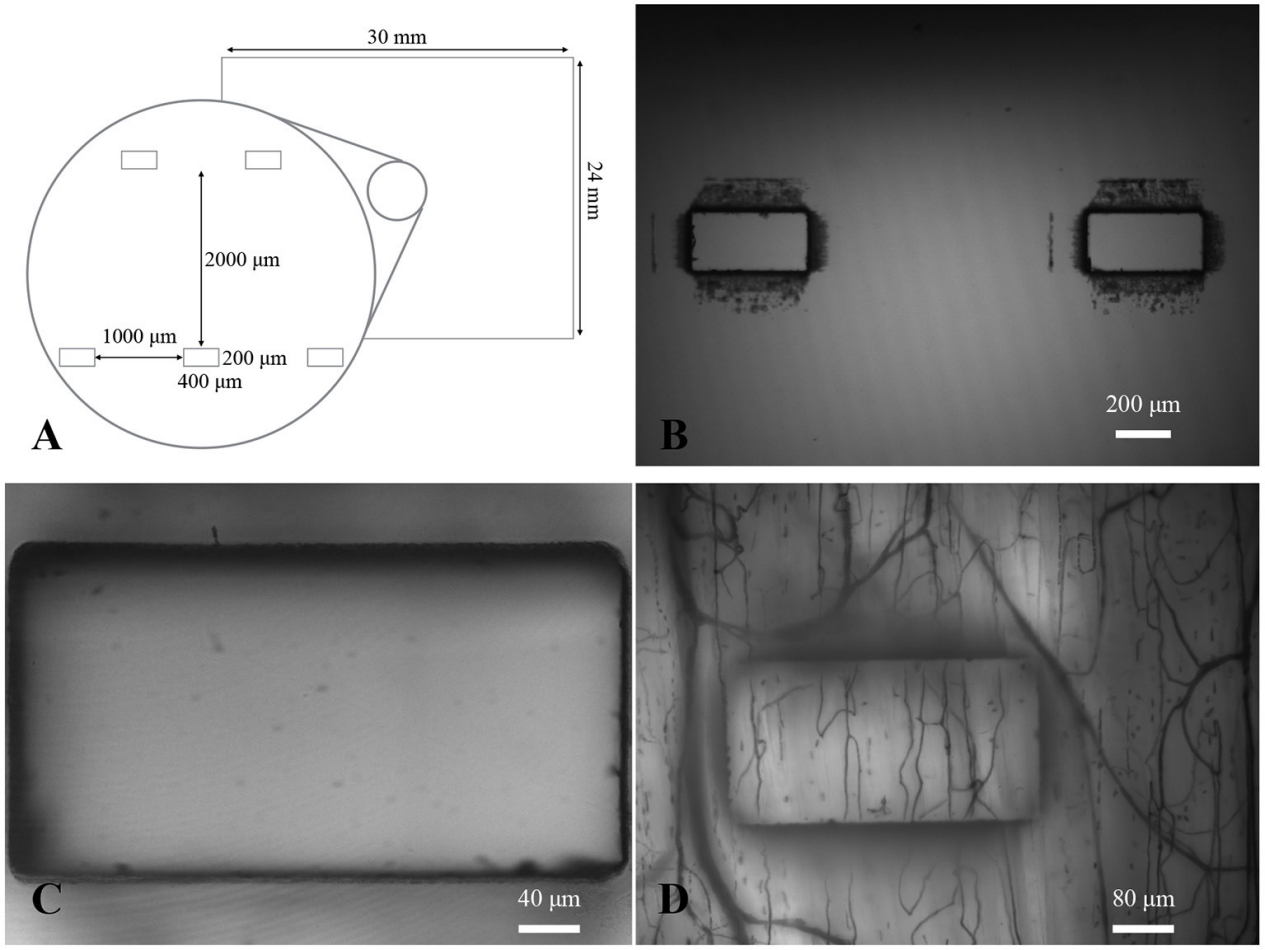
Gas exchange window design. **(A)** Diagram of the design of the gas exchange windows. **(B)** A 4X micrograph showing two of the exchange windows centered in the field of view. Dark markings from laser machining can be seen around the edges of each window. **(C)** A 20X micrograph of an exchange window focused on the edge closest to the objective. **(D)** A 10X functional image of the minimum intensity values over time with dark lines showing location of flowing capillaries and larger micro vessels (as well as outline of the window).

**Figure 4:**
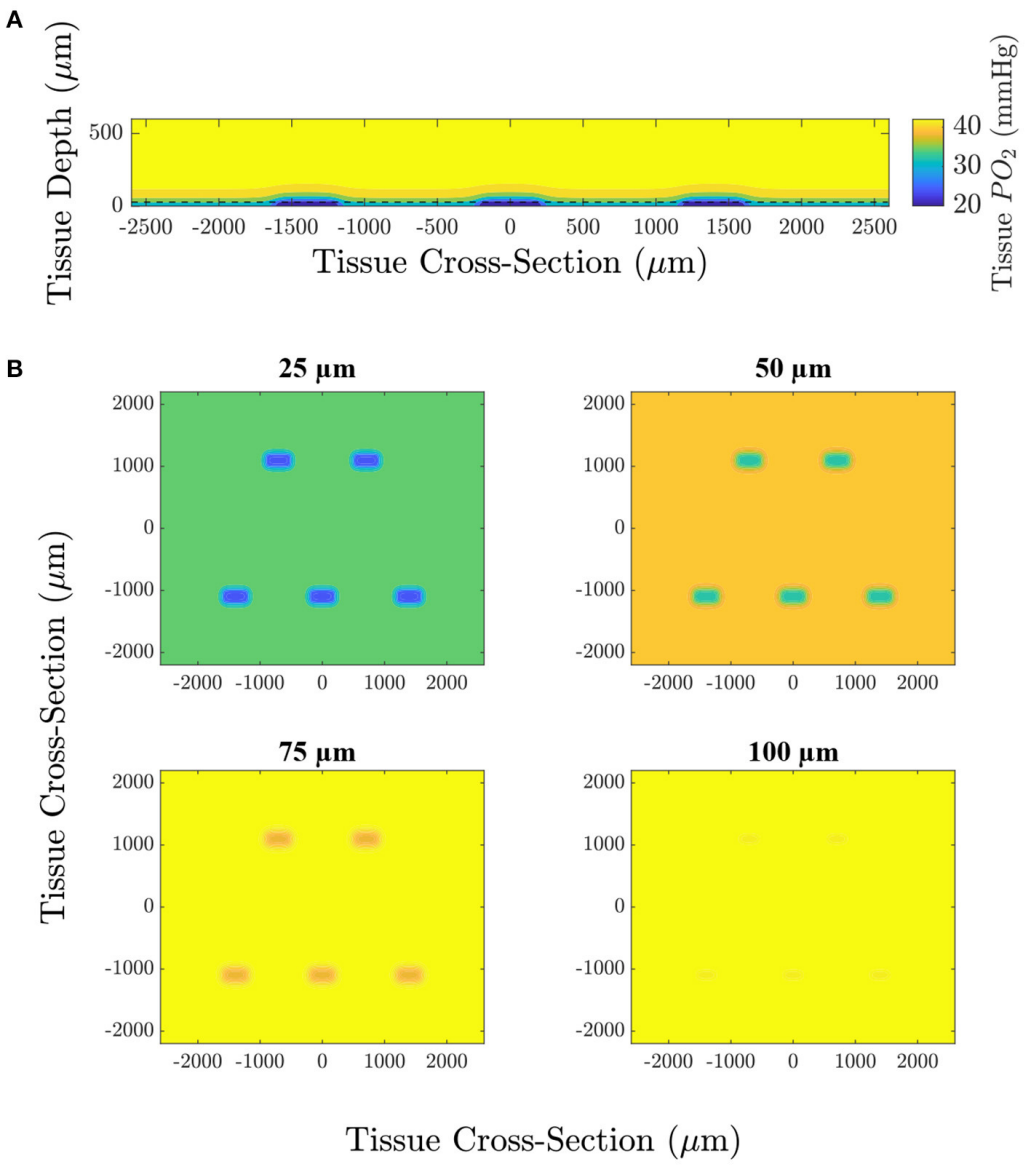
Computational simulation predicting the tissue PO2 resulting from diffusional exchange between the tissue and gas exchange chamber in response to a low O2 challenge. Results are presented as a contour map of the steady-state O2 distribution in the tissue around the gas exchange windows with a 25 μm thick PDMS layer. **(A)** Section through the long axis of the window oriented normal to the imaging plane of the microscope. The dashed line indicates the position of the top of the PDMS layer. **(B)** Sections oriented with the imaging plane at depths of 25, 50, 75, and 100 μm from the surface of the glass slide.

Five gas exchange windows were patterned into glass slides to facilitate positioning of the muscle relative to the exchange window (Figure 3). Windows were designed to be 200 by 400 μm. The spacing of the windows was chosen to allow for regions between the windows that are unaffected by the change in O2. This aim was supported by the results of our mathematical model; see Figure 4. Dark markings from the laser cutting process can been seen around the edges of the windows; this is due to the laser fabrication process increasing light scatter near the cut edges. It can be noted that these marks only appear on one side of the glass slide. We chose the non-marked side to be in contact with the muscle to ensure that the markings are out of the focal plane when focused on the muscle; this can be seen in Figures 3C,D.

**Figure 5:**
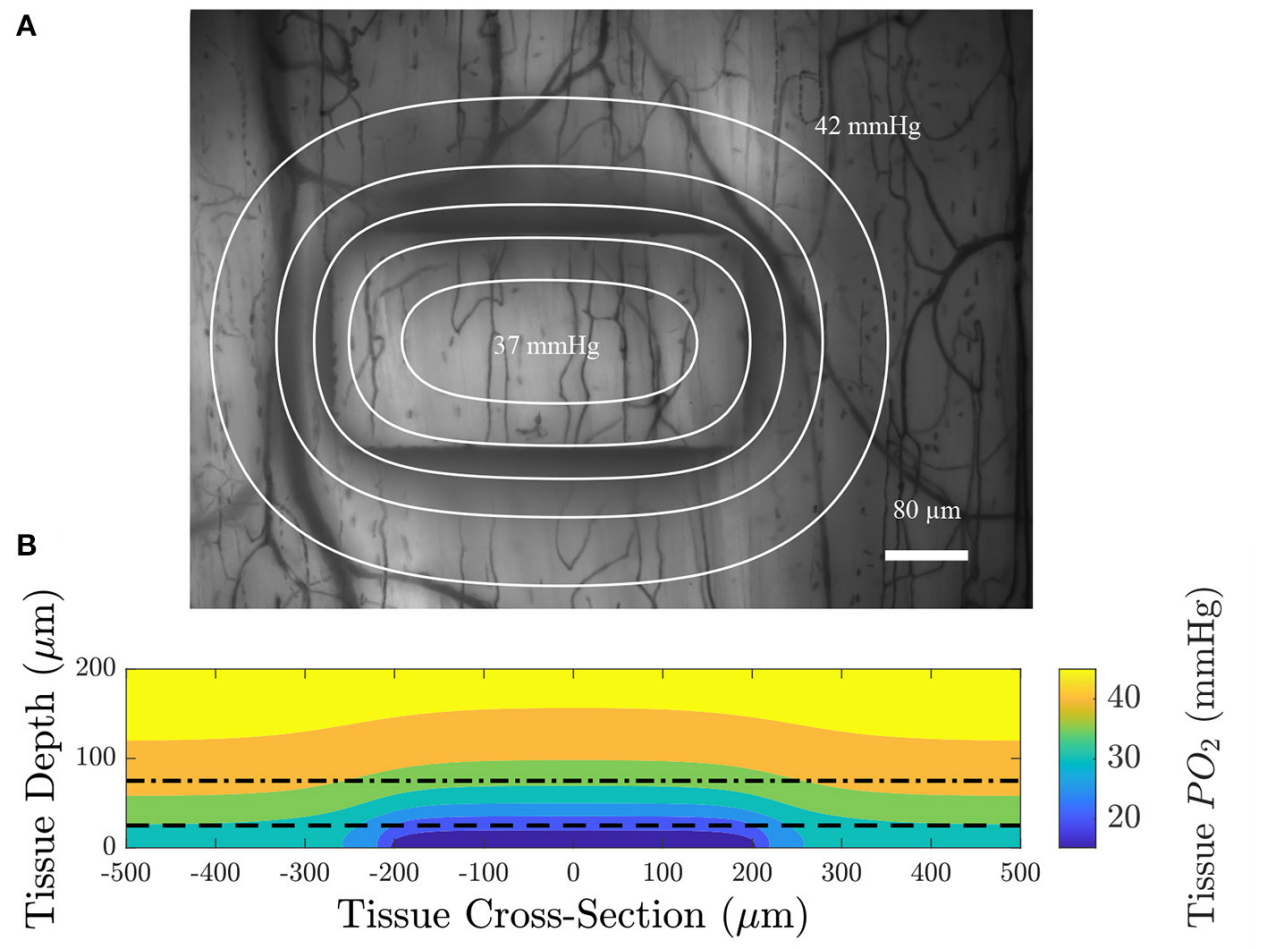
**(A)** An intravital video microscopy functional image of the minimum pixel intensity over time with contour lines displaying constant PO_2_ at a depth of 75 μm from the top surface of the glass slide (dotted dashed line in **B**); each contour is spaced 1 mmHg apart. **(B)** A colormap of the oxygen distribution as a function of depth in the tissue for a section through the long axis of the window; the dashed line indicates the location of the top of the PDMS layer and the dotted dashed line indicates the position of the imaging plane. The model predicts that the area affected by low oxygen challenges imposed by the gas exchange platform extend over an approximate area of 614 by 434 μm.

We used a model of O2 transport that was modified from that used in Goldman (2008) and Ghonaim et al. (2011) to predict the distribution of O2 in the tissue and to determine the extent to which the perturbation penetrates the tissue. It should be noted that this model only considers diffusion, neglecting convective effects caused by RBCs transporting O2 which would further limit the extent of the perturbation into the tissue. Figure 4 shows the steady-state PO2 distribution for a low (2%) O2 challenge in five different planes for a large section of tissue. PO2 distribution in a plane normal to the imaging plane of the microscope is given to show the depth of penetration of the perturbation. The largest predicted depth of penetration occurs in the center of the window, reaching a depth of 130 μm from the tissue surface. Additionally, the radial extend of the decrease in oxygen is shown by the PO2 distribution at the tissue surface and at depths of 25, 50, and 75 μm from the exchange window surface. The decrease in tissue oxygen is predicted to extend a maximum of 109 μm from the the edge of the window in the direction of the long axis of the window and 117 μm in the direction of the short axis of the window. Due to the high solubility of O2 in PDMS, tissue depths below ~50 μm from the glass slide (75 μm from the surface of the glass slide) are predicted to experience non-local changes in O2. This effect would be exacerbated for thicker PDMS layers. Figure 5 shows the model predictions of PO2 close to the window, with the first panel displaying contour lines over a functional microscopy image displaying the anatomical structure of the vasculature in the tissue. At this depth, the low O2 condition is predicted to only impose SO2 changes in capillaries directly overlying the exchange window.

**Figure 6:**
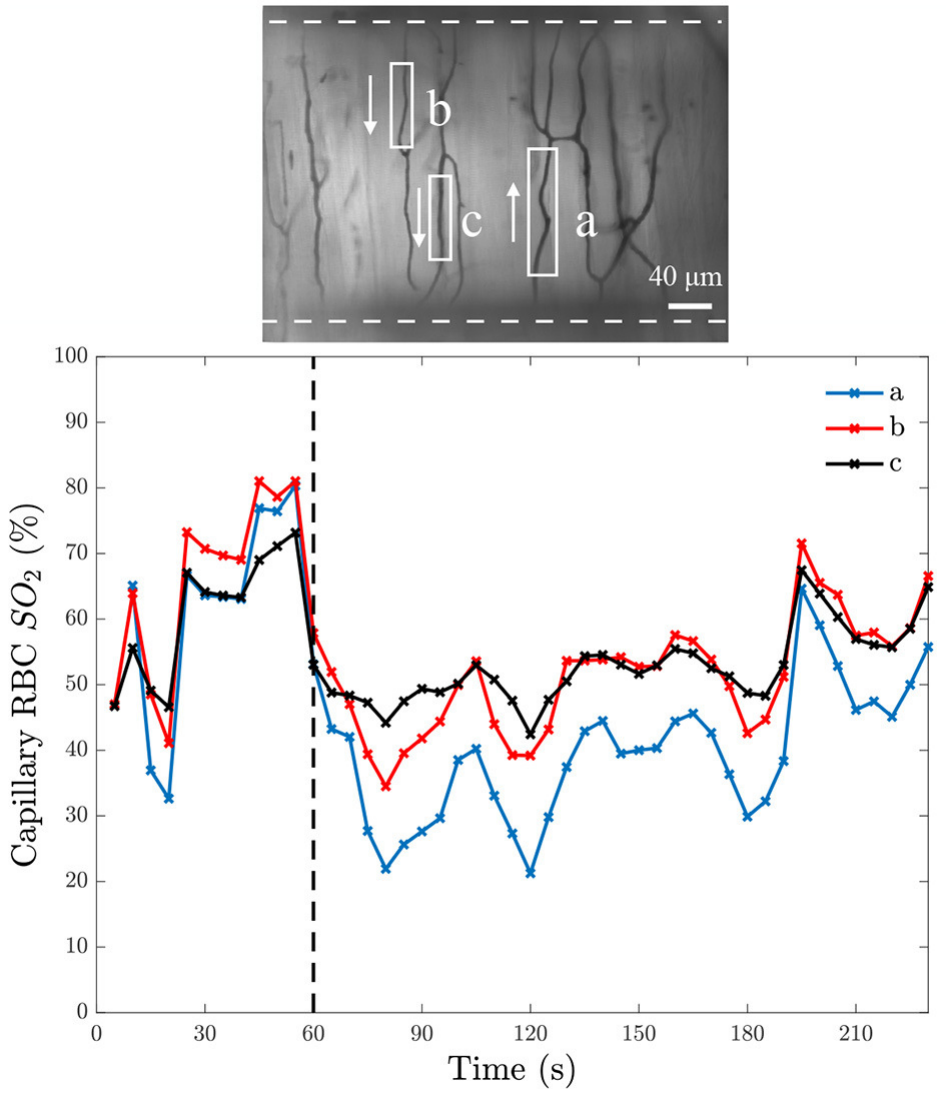
Capillary red blood cell oxygen saturation in response to a step change in O2. The bottom panel shows mean capillary red blood cell (RBC) oxygen saturation (SO2) of 5-s bins from three separate capillaries in the same field of view over time; the time at which O2 was changed is indicated with the black dashed line. The field of view from which the measurements were taken is represented by the intravital microscopy functional image of the minimum pixel intensity over the duration of the video shown in the top panel. The analyzed capillaries in the top panel are identified by white boxes, with arrows to indicate the direction of RBC flow, and the white dashed line indicates the position of the O_2_ exchange window.

To verify that the exchange window is affecting RBC SO2, we performed step changes in chamber [O2] and measured the resulting RBC SO2. At baseline, the gas composition contained 5% O2, 5% CO2, and balance N2. After 1 min, the gas composition was changed to 2% O2, 5% CO2, and balance N2. An example of a step change for multiple capillaries in a single field of view is shown in Figure 6. After the drop in gas chamber O2, the SO2 drops rapidly then steadily increases. This increase can be explained by the increased RBC flow rate in response to the low O2. It can also be noted that the trend is similar for all capillaries in the field of view overlying the micro-outlet.

**Figure 7:**
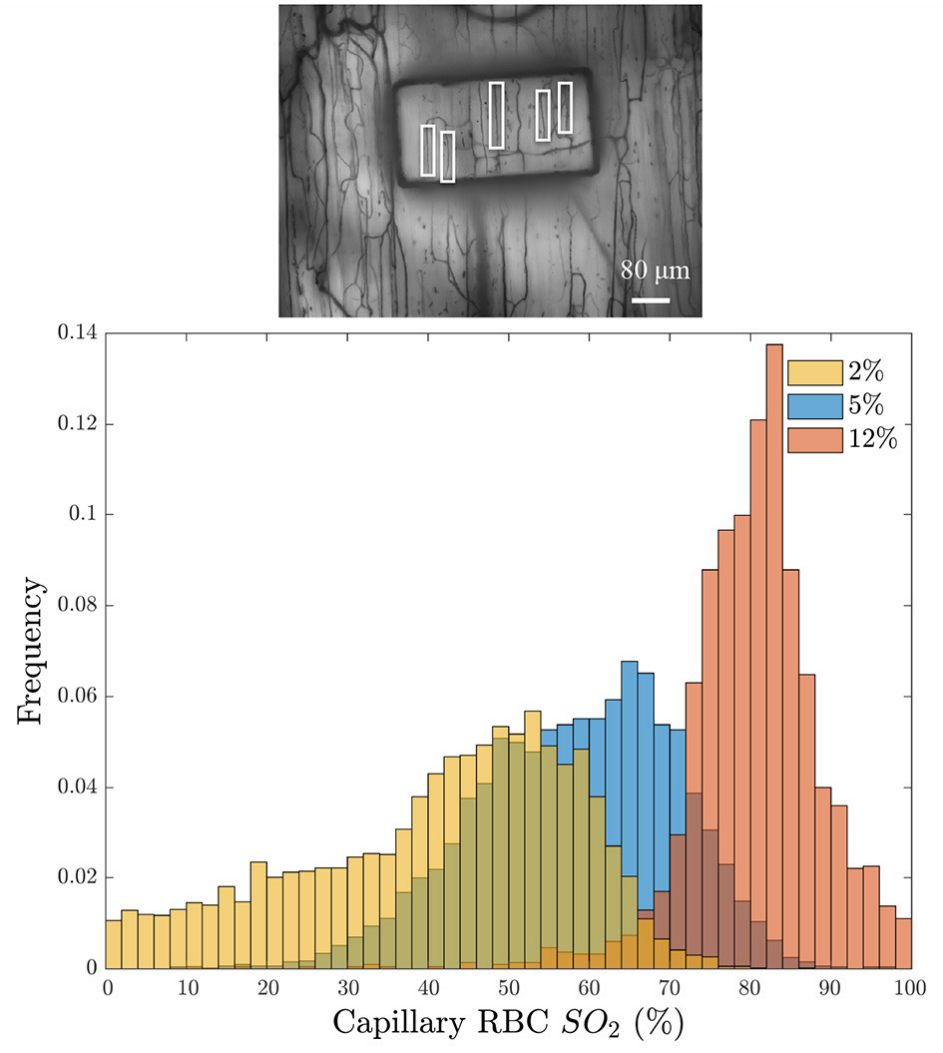
**(Bottom)** Distribution of the average capillary red blood cell oxygen saturation (Capillary RBC SO2) binned into 5-s intervals in response to three window O2 levels: 2% (orange), 5% (blue), and 12% (red). Histogram bins are partially transparent to show the overlap. The minimum intensity functional image of the analyzed field of view is given above the histogram **(Top)**; the analyzed capillaries are indicated with white boxes.

A further demonstration of the desaturation capabilities of this device are shown in Figure 7. This figure shows the distribution of capillary SO2 values when the window O2 is set to 2, 5, and 12% (5% CO2 and balance N2). These results demonstrate the large variations in RBC SO2 experienced in the microcirculation, even when subjected to variations in window O2. The variations are due to the variability in RBC supply rate between vessels, which vary from approximately 2 RBC/s to 40 RBC/s in this example field of view.

**Figure 8:**
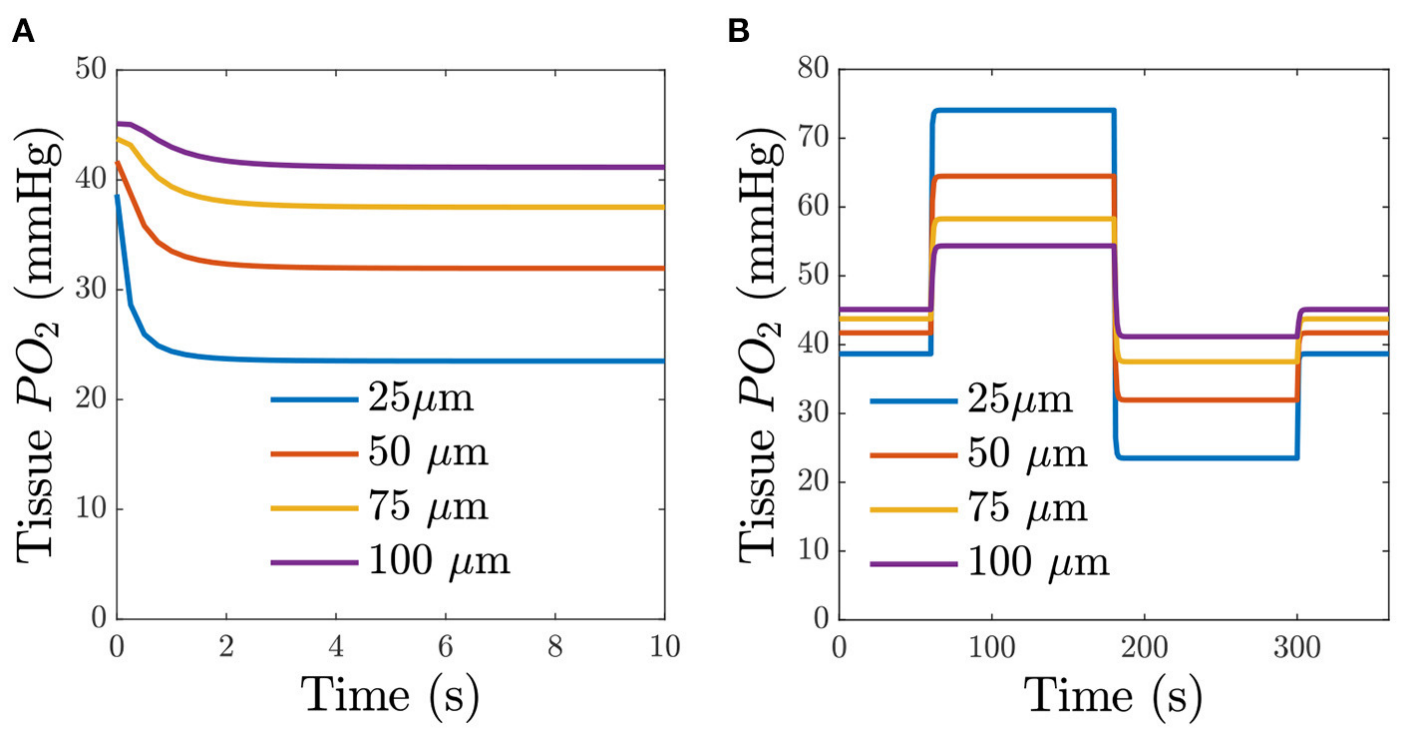
Predicted time-dependent changes in tissue O2. **(A)** Predicted tissue PO2 for a step change in chamber O2 for different depths from the glass slide. **(B)** Predicted tissue PO2 in response to a square wave in chamber O2. The square-wave consisted of 1 min of 5% O2, 2 min of 12% O2, 2 min of 2% O2, and 1 min of 5% O2. The legend indicates the depth from the surface of the exchange window.

The computational model was also used to verify how quickly changes in chamber O2 affect tissue O2 at varying depths in the tissue. The model assumes the gas composition at the window changes instantly; thus the model is determining the temporal diffusion limitation. Figure 8A shows the simulation results for a step change in O2 from 5 to 2% centered in the window at varying tissue depths. The results of a square wave in O2 are shown in Figure 8B. The simulation demonstrates that diffusion reaches steady-state within 3 s of the the step change.

**Figure 9:**
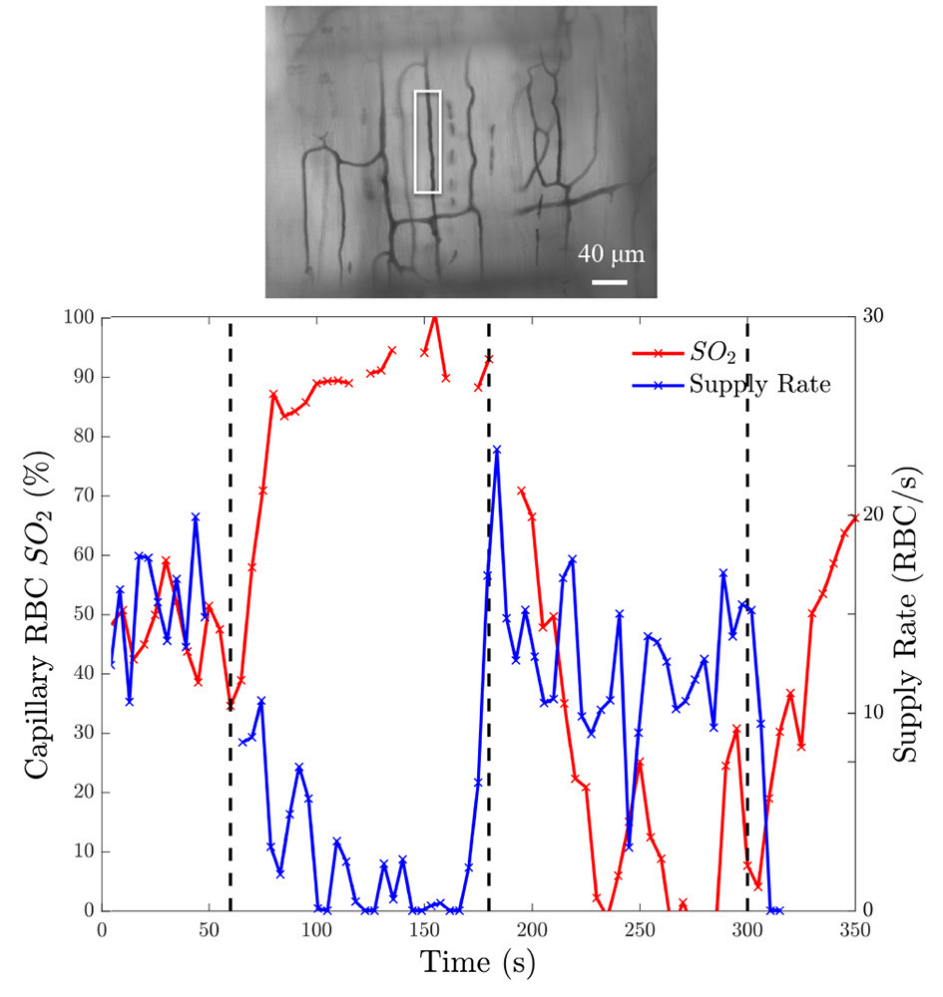
Oxygen saturation and supply rate change in response to square wave oxygen challenge from a baseline of 5% O2 followed by 2 min of 12%, 2 min of 2%, and 1 min of 5% with 5% CO2 and balance N2. **(Bottom)** Induced SO2 changes is given on the left ordinate axis and the corresponding change in RBC supply rate is given on the right ordinate axis. The black dashed lines indicate the time at which the chamber O2 was changed. The analyzed capillary is indicated with a white box in the minimum intensity functional image above the figure **(Top)**.

**Figure 10:**
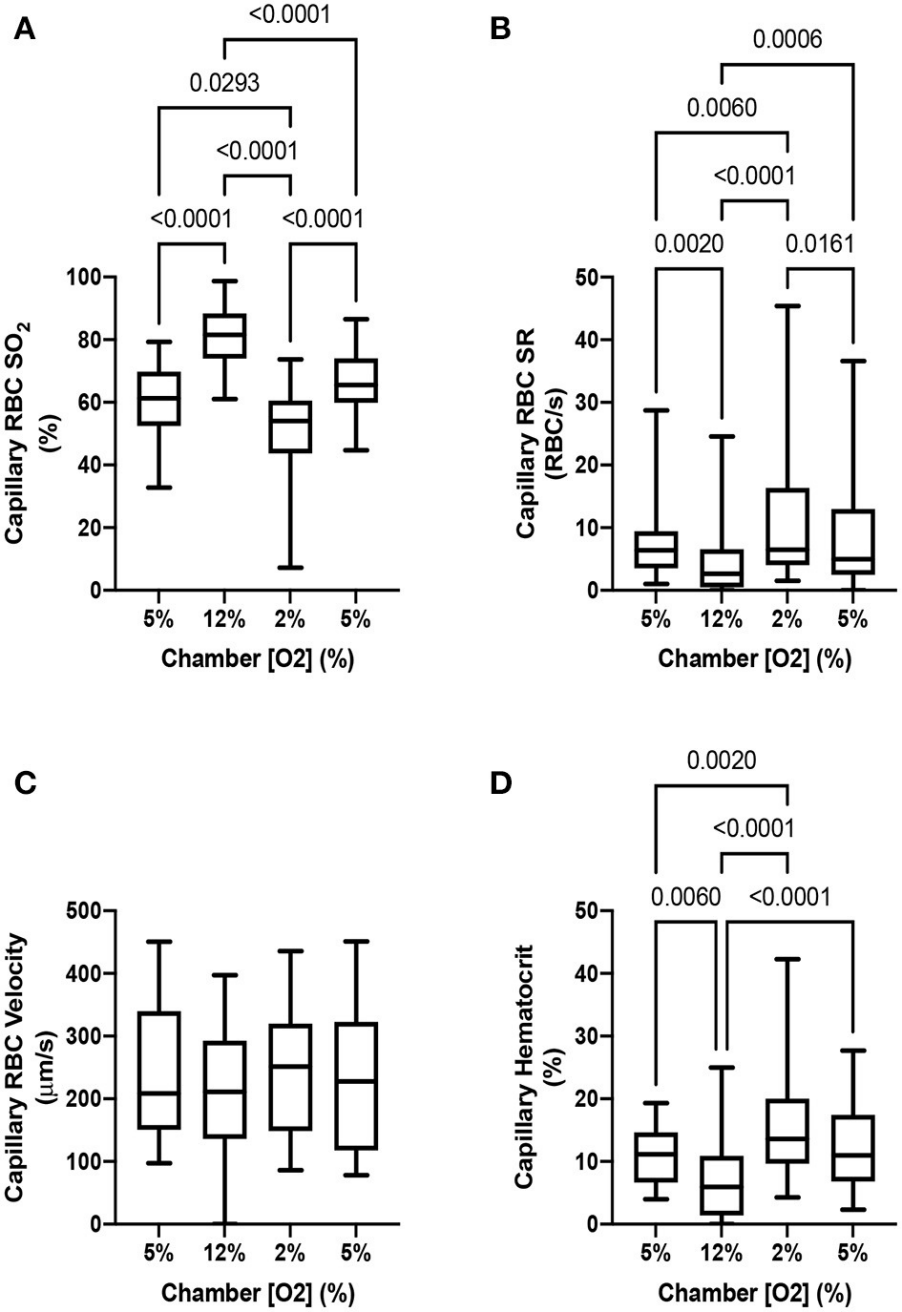
Capillary hemodynamics and oxygen saturation responses in capillaries directly overlying exchange windows of the modular gas exchange platform. The platform imposed a square wave oxygen challenge from a baseline of 5% O2 chamber oxygen concentration ([O2]) followed by 2 min of 12%, 2 min of 2%, and 1 min of 5% with 5% CO2 and balance N2. **(A)** shows the capillary red blood cell (RBC) oxygen saturation (SO2) in the last 30s of each imposed chamber [O2] across 14 fields in 4 animals (*n* = 24 capillaries). The resulting blood flow response to the change in SO2 is illustrated by the capillary RBC supply rate (SR) and hematocrit changes shown in **(B,D)** (*n* = 32 capillaries). Changes in capillary RBC velocity **(C)** were not found to be significant between any chamber [O2] (*n* = 29 capillaries). *p* values based on Tukey's multiple comparisons test after significant repeated measures ANOVA (capillary RBC SO2), and Dunn's multiple comparisons test after significant Friedman test (capillary RBC SR and hematocrit) are indicated in the figure with a *p* < 0.05 considered to be significant. Box and whisker plots show minimum, median, maximum, and associated quartiles.

To demonstrate that the device can induce flow rate responses, we measured both RBC SO2 in selected in-focus capillaries as well as RBC supply rate in response to a square wave change in chamber [O2]. The square wave consisted of 1 min of 5% O2 followed by 2 min of 12%, 2 min of 2%, and 1 min of 5% with static 5% CO2 and balance N2. Representative responses from a single field of view from a the square wave change in platform [O2] is shown in Figure 9. Responses from square-wave oscillations of capillaries directly overlying the exchange window were determined based on measurements in the last 30s of each step from 14 fields of view across 4 animals and are shown in Figure 10. Capillary RBC SO2 data was not found to significantly deviate from assumed normal distributions and therefore parametric tests were applied. RBC SO2 of capillaries directly overlying the window at the initial 5% chamber [O2] was 60.4 ± 12.7% and was found to be significantly different than capillary RBC SO2 at chamber concentrations of 12%, 81.3 ± 9.60%, and 2%, 50.5 ± 16.16% (*p* < 0.0001 and 0.0293 respectively, *n* = 24 capillaries). Data sets for capillary velocity, hematocrit, and supply rate were all found to deviate significantly from the assumed normal distribution and non-parametric tests were applied accordingly. The imposed chamber [O2] caused significant changes in capillary hematocrit from the initial 5% chamber [O2], 11.1 ± 4.59%, compared to chamber [O2] of 12%, 7.0 ± 5.87% (*p* = 0.0060), and 2% chamber [O2], 15.6 ± 8.63% (*p* = 0.0020, *n* = 32 capillaries). Similarly, changes in chamber [O2] caused flow changes as measured by capillary RBC SR between the initial 5% condition, 7.9 ± 6.43, vs. 12%, 4.4 ± 5.4 cells/s (*p* = 0.0020), as well as at the 2% [O2], 11.5 ± 10.69 (*p* = 0.0060, *n* = 32 capillaries).

**Figure 11:**
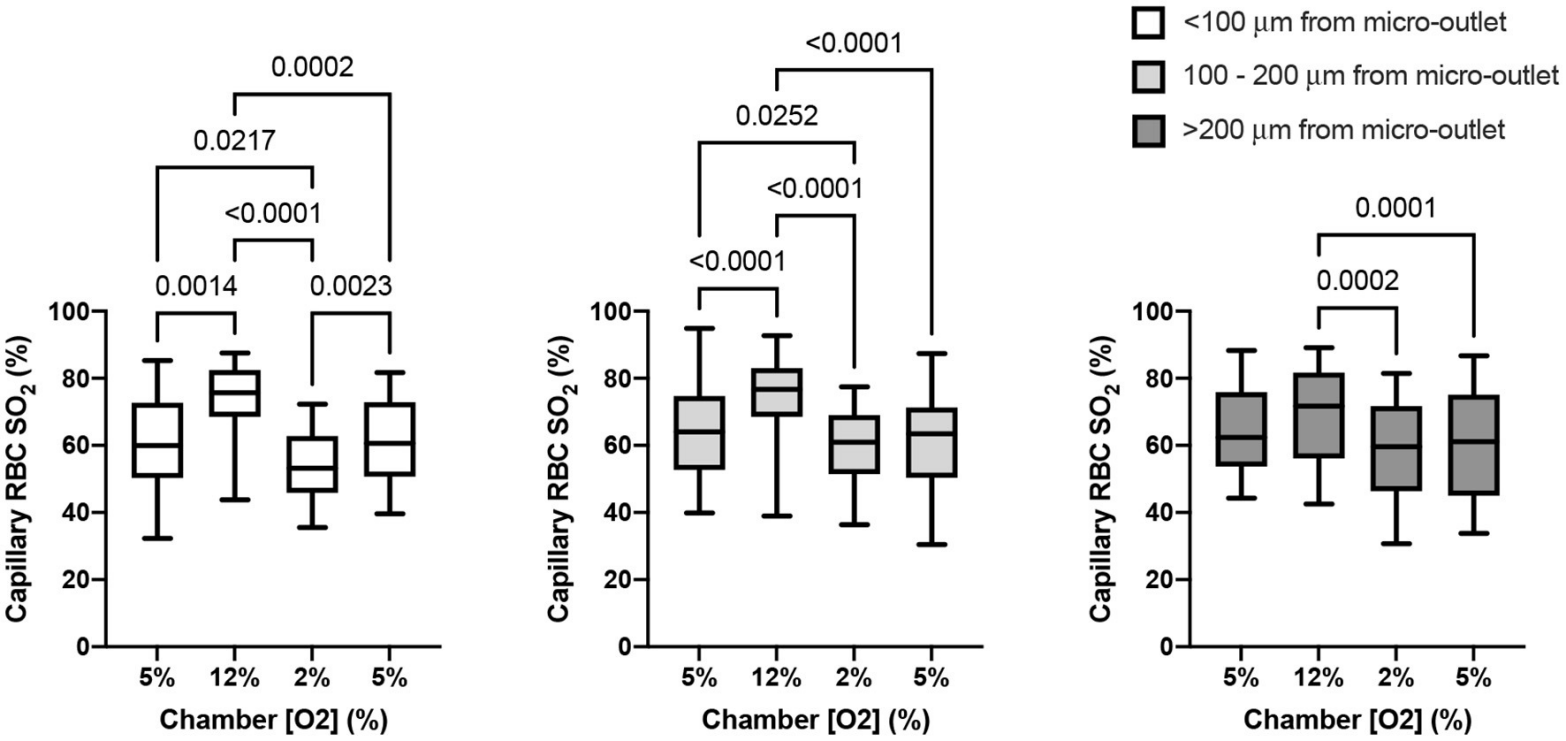
Capillary oxygen saturation responses in capillaries at various distances from the exchange windows of the modular gas exchange platform. The platform imposed a square wave oxygen challenge from a baseline of 5% O2 followed by 2 min of 12%, 2 min of 2%, and 1 min of 5% each with 5% CO2 and balance N2. Each panel shows the capillary red blood cell (RBC) oxygen saturation (SO2) in the last 30s of each imposed platform oxygen concentration across 14 fields in 4 animals. The left panel shows oxygen saturations in capillaries within < 100 μm from the exchange window (*n* = 17 capillaries). The middle panel shows mean capillary RBC SO2 in vessels between 100 and 200 μm from the window (*n* = 27 capillaries), and the right panel shows responses in capillaries > 200 μm from the edge of the exchange window (*n* = 19 capillaries). With increasing distance the magnitude of the SO2 change diminishes, though significant changes in SO2 imposed by the low oxygen condition in the platform were detectable beyond 200 μm. *p* values based on Tukey's multiple comparisons test after significant repeated measures ANOVA are indicated in the figure with a *p* < 0.05 considered to be significant. Box and whisker plots show minimum, median, maximum, and associated quartiles.

The capability of the oxygen exchange platform to alter RBC SO2 in capillaries at a distance from the exchange window was assessed for all in focus vessels < 100 μm from the window, 100–200 μm from the window, and in vessels > 200 μm from the window (Figure 11). Capillary RBC SO2 data for each grouping of vessels outside of the window was not found to significantly deviate from assumed normal distributions. For vessels < 100 μm outside the window, oscillations in chamber [O2] caused significant changes in capillary SO2 at 12% [O2], 74.5 ± 10.91% (*p* = 0.0014), and 2% [O2], 53.9 ± 10.84% (*p* = 0.0217), compared to the mean capillary SO2 of 60.4 ± 14.17% at the initial 5% condition (*n* = 17 capillaries). Similarly, significant changes in SO2 were found in capillaries 100–200 μm from the window at 12% [O2], 73.9 ± 12.24% (*p* < 0.0001), and 2% [O2], 59.5 ± 11.68% (*p* = 0.0252), compared to the capillary SO2 of 64.0 ± 13.32% at the initial 5% condition (*n* = 27 capillaries). In vessels > 200 μm from the window no significant change in capillary SO2 compared to the initial 5% [O2] was detected (63.6 ± 12.48%), although SO2 was significantly different between the 12 and 2% conditions, 68.6 ± 14.22% and 57.8 ± 16.23% (*p* = 0.0005, *n* = 20 capillaries). No robust changes in mean capillary hemodynamic measures were noted for vessels at a distance from the window grouped by the three distance delineations described above (data not shown).

## 4. Discussion

In this study, we developed a modular gas exchange platform to deliver a localized gas composition to the surface of externalized EDL muscle tissue for use in intravital microscopy studies. Our model predicts that the platform is able to change RBC SO2 in capillaries within a localized area of approximately 614 by 434 μm (Figure 5). The changes in capillary RBC SO2 were demonstrated both experimentally (Figures 6, 7, 10, 11) and computationally (Figure 5); the later predicts that the effect of the perturbation extends to a maximum of 117 μm beyond the edge of the window which is an important and novel insight resulting from the present work. This diffusive spread of PO2 within the exchange membrane has not previously been reported as earlier studies did not include the exchange membrane itself as an element of the model (Ghonaim et al., 2011, 2013). Additionally, our experimental results demonstrate that the changes in tissue PO2 propagate even further than our mathematical model predicts with measurable changes in capillary SO2 extending beyond 200 μm (Figure 11). The discordance between our model's prediction and the experimental result is likely due to a mismatch in assumed model parameters, particularly muscle oxygen consumption; our present model also does not consider the specific microvascular geometry and spatial location of vessels within the volume which is likely to contribute to the difference between the predicted change in tissue PO2 and the observed SO2 changes. This is an important consideration for future studies seeking to spatially constrain oxygen challenges to specific vascular structures or regions of interest. The computational model also predicts that O2 diffusion into the tissue will reach steady-state within 3 s of changing the chamber O2. As with the distance to which the low oxygen challenge extends into the tissue, the prediction for this time transient may be impacted by the location of individual blood vessels within the tissue, for which the model does not account.

In previous work, we used a smaller gas exchange window to induce capillary RBC SO2 changes (Ghonaim et al., 2011). Ghonaim et al. suggested that not enough capillaries were stimulated to elicit a flow response (Ghonaim, 2013; Ghonaim et al., 2013). This is supported by the use of a larger gas exchange window to induce RBC SO2 changes in more capillaries to which the vasculature responded (Ghonaim, 2013). In the current study, we used a window size (400 × 200 μm rectangular window) that was larger than that used in Ghonaim et al. (2011) (100 μm diameter circular window) but smaller than that used in Ghonaim (2013) (1.000 × 200 μm rectangular window). Our present configuration was capable of imposing significant changes in capillary SO2 and concomitant hemodynamic responses in vessels directly overlying the exchange window (Figure 10). The changes in SO2 in both (Ghonaim et al., 2011; Ghonaim, 2013) were consistent with those in our chamber. This finding supports the hypothesis that the ATP release signal is additive since we are affecting more capillaries than in Ghonaim et al. (2011).

Various studies in the literature have been successful in imposing changes in RBC SO2 both *in vivo* and *ex vivo* (Duling, 1972; Pittman and Duling, 1973; Hutchins et al., 1974; Fredricks et al., 1994; Welsh et al., 1998; Zhu et al., 1998; Frisbee and Lombard, 2002; Frisbee et al., 2002). One approach is to alter the inspired O2 levels as in Zhu et al. (1998), resulting in changed RBC SO2, though this may result in systemic hyper/hypoxia (Jackson, 2016). Another approach involves using superfusion solutions with different gas compositions to bathe the tissue in order to control the surface O2 levels (Frisbee and Lombard, 2002; Frisbee et al., 2002). While this method confines the changes in O2 to the tissue being studied, due to the low solubility of O2 in water, superfusion solutions have a limited ability to change SO2 and lacks spatial specificity, particularly as it pertains to the levels of vasculature being affected. Additionally, our current approach is able to produce a more rapid change in PO2 compared to those using superfusion solutions. For these reasons, gas exchange chambers may be more advantageous compared to other approaches in the investigations of localized O2 regulation.

Despite the many benefits of the approach employed in this work, there are a few challenges that are worth noting. Firstly, due to the micro-outlet patterned in the glass, the tissue viewed through the window opening is in a different focal plane than the surrounding tissue at the same depth of focus. Because of this, it is not possible to focus on capillaries in and out of the window at the same tissue depth simultaneously. However, due to the excellent optical clarity outside the window, it is possible to focus on capillaries outside of the window, enabling measurement of unperturbed hemodynamics and SO2 levels in the tissue at a sufficient distance from the window.

Another challenge associated with this experiment is placement of the muscle over the exchange windows. This requires careful manipulation of the muscle in order to place the muscle over the windows such that the imposed perturbation would affect capillary modules of interest. This is an important consideration since moving the muscle multiple times may increase the likelihood of the muscle becoming stressed and impairing the vasculature to respond to changes in O2. Including additional windows closer together would increase the probability of one of the windows being over an area of interest in the muscle, though care must be taken to ensure that the other windows do not interact with vessels in the region being observed.

Our device is well-suited to studying oxygen regulation at the microvascular level. For example, our method could be used to interrogate whether changes in capillary RBC distribution are due to passive rheology (i.e., bifurcation law) or if there are other active mechanisms in place, such as pericytes that respond to changes in O2 to control flow in capillaries. Indeed, Ellis et al. (1994) showed that as supply rate increases, the distribution of flow becomes more homogenous. To determine if this effect is purely due to passive rheology, one could position the window over capillaries fed from the same arteriole to identify if their distribution of flow rates among the capillaries remains constant in response to oxygen. Further, this platform could be used to determine if stimulating capillaries in one capillary module affects adjacent modules connected to the same feed arteriole. Such an experiment could help support the SO2-dependent ATP release from RBC hypothesis. Conversely, if the O2 sensor is located in the extravascular space rather than the RBCs, the O2 exchange platform could further be used to stimulate areas of the muscle that lack capillaries to investigate the presence of a tissue sensor.

Additionally, this approach could be used in *ex vivo* and *in vitro* studies where the control of gas composition needs to be locally confined. For example, this gas exchange platform could be used in conjunction with a microfluidic device to desaturate flowing RBCs as suggested in Sove et al. (2013) and Sové et al. (2016). As proposed in these studies, such a device could probe the dynamics of the ATP release mechanism if it is indeed caused by RBC desaturation.

In summary, we have developed a modular gas exchange platform capable of causing local changes in capillary RBC SO2 and stimulating corresponding responses in capillary hemodynamics. We have also shown that these changes are consistent with the ATP release hypothesis that multiple capillaries need to be stimulated in order to elicit a microvascular flow rate response. While our device stimulates a large enough region to obtain a flow response, it is also localized enough that we will be able to probe specific spatial regions in the microvascular bed, and the device's excellent optical clarity allows for direct observation of the response both in the stimulated, as well as neighboring regions. This tool offers exciting possibilities to study microvascular oxygen regulation, and may aid to definitively determine the location of the elusive oxygen sensor.

## Data Availability Statement

The data supporting the conclusions of this article will be made available by the authors, without undue reservation.

## Ethics Statement

The animal study was reviewed and approved by University of Western Ontario's Animal Care and Use Committee.

## Author Contributions

This study was conceived and designed by RS, CE, and GF. The modular gas exchange chamber was designed by RS, DH, and HN aided in the fabrication. RS and SM collected the *in vivo* experimental data. RS wrote the mathematical model and generated the resulting simulation data. Data analysis and interpretation was done by RS, SM, CE, and GF. RS wrote the manuscript with input from all authors. Critical revision was done by RS, CE, and GF.

## Conflict of Interest

The authors declare that the research was conducted in the absence of any commercial or financial relationships that could be construed as a potential conflict of interest.
